# Sociability between invasive guppies and native topminnows

**DOI:** 10.1371/journal.pone.0192539

**Published:** 2018-02-14

**Authors:** Morelia Camacho-Cervantes, Alfredo F. Ojanguren, Omar Domínguez-Domínguez, Anne E. Magurran

**Affiliations:** 1 Centre for Biological Diversity, University of St Andrews, St Andrews, Fife, United Kingdom; 2 Facultad de Biología, Universidad Michoacana de San Nicolás de Hidalgo, Morelia, Michoacan, Mexico; Uppsala Universitet, SWEDEN

## Abstract

The role of interspecific social interactions during species invasions may be more decisive than previously thought. Research has revealed that invasive fish improve their foraging success by shoaling with native Mexican species, and potentially increase the chances of invasion success. However, do native individuals tend to associate with invaders as well? We tested the hypothesis that the twoline skiffia (*Neotoca bilineata*) and the Lerma livebearer (*Poeciliopsis infans*), both native endemic Mexican topminnows, will associate with guppies, a notorious invasive species present in Mexico. Our investigation shows that guppies, twoline skiffias and Lerma livebearers have a mutual tendency to associate with each other. Although there is a marked tendency to shoal with heterospecifics in this system, shoaling partners do not necessarily benefit equally from the association. Further research on invasive-native social interactions is needed to promote our understanding of potential facilitation by natives.

## Introduction

Animals associate when the “joint project” (e.g., finding food, exploring surroundings or avoiding predators) delivers benefits to all the individuals involved [1]. Although these associations occur most often among conspecifics, temporary aggregations that include different species have been observed across taxonomic groups [2, 3]. Some birds, for example, choose habitat patches based on the presence of resident individuals of a different species [4]. Similarly, newts are able to locate breeding ponds using toad calls, and even show preference for species that indicate more suitable areas [5].

In the wild, individual fish actively choose when to join a mixed species shoal based on size and species involved [6]. Other drivers such as phenotypic similarity, group size [7], nutritional state of members [8] and parasite load [9] may also be involved. In the case of associations between fathead minnows (*Pimephales promelas*) and brook sticklebacks (*Culaea inconstans*), shoal choice decisions are influenced by perceived levels of predation risk and competition for food [10]. These differences might be related to the differential vulnerability of the two species, since sticklebacks are armoured but minnows are not.

Invaders are often introduced at low densities, making them vulnerable to the disadvantages of being part of a small group, such as being less effective when avoiding predators or taking longer to locate food and spending less time feeding [11]. Sociable species could avoid local extinction if they are able to join groups of more abundant species that facilitate group tasks. Heterospecific associations could improve chances of survival when low population sizes result in reduced fitness [12].

Interactions between natives and invaders are more often thought to be negative (*e*.*g*. competition or predation) as it is believed that native communities tend to resist invasion [13]. However, positive heterospecific interactions play an important but often unrecognised role in the invasion process by facilitating the establishment of invaders [14]. Environments where positive interactions occur between invaders and the species they encounter might be at higher risk of invasion [15, 16].

The Trinidadian guppy (*Poecilia reticulata*) is a shoaling species that has invaded freshwater habitats throughout the world [17]. Native to Trinidad, Guyana, Venezuela and Surinam [18], guppies have been introduced deliberately as mosquito control or accidentally as a consequence of the aquarium trade [19]. In México, guppies have successfully established populations in the Lerma-Santiago river system, the main basin of the Mexican High Plateau, a watershed noted for its high levels of endemicity [20]. Guppies have been found to cause declines of local populations of the endemic Goodeinae topminnows [21]. The negative effects of invasive guppies range from competition for food to even sexual harassment of goodeinae females by male guppies [22].

It has been demonstrated that guppies tend to associate with native species and gain benefits from doing so [23–25]. However, it is unknown whether natives show a mutual tendency to associate with guppies or if heterospecific associations occur due to the imposition of one species over the others. The aim of this study is to assess the tendency of native species to conform heterospecific shoals that include invaders. We tested the hypothesis that two native Mexican topminnows: twoline skiffia (*Neotoca bilineata*) and Lerma livebearer (*Poeciliopsis infans*) are willing to associate with heterospecifics as guppies do. Willingness of native species to interact with invasive species could be a key environmental trait increasing the local vulnerability to invasion by helping founding individuals attenuate disadvantages of being part of a small population.

## Materials and methods

We measured the tendency to associate with conspecific and heterospecific shoals of twoline skiffia, Lerma livebearer and guppies. Experiments were carried out at the UMSNH in México (March and April 2012). Fish were collected from the wild in three separate locations. Guppies were collected in Maravatío (19° 53’ 01” N, 100° 26’ 50” W), twoline skiffias in Cuitzeo Lake (19° 54’ 27” N, 101° 04’ 32” W) and Lerma livebearers in La Mintzita (19° 38’ 43” N, 101° 16’ 29” W); all in Michoacán State. In the sites where we collected each species none of the other two could be found, thus no fish had previous experience with individuals of the other two species used in this experiment. However, there are water bodies in the Lerma-Santiago river system where these species can be found sympatrically (Domínguez-Domínguez, pers. com.). In addition, focals were kept separated by species and from shoal individuals for over two weeks to avoid familiarity effects [26]. Fish were kept in stock tanks (50 L) filled with aged tap water that was continually filtered, aerated and treated with Stress Coat^®^. Temperature ranged from 19°C to 23°C and photoperiod was approximately 13L: 11D from 700 to 2000. Fish were fed commercial flake food daily at least one hour before and immediately after the observations.

**Fig 1:**
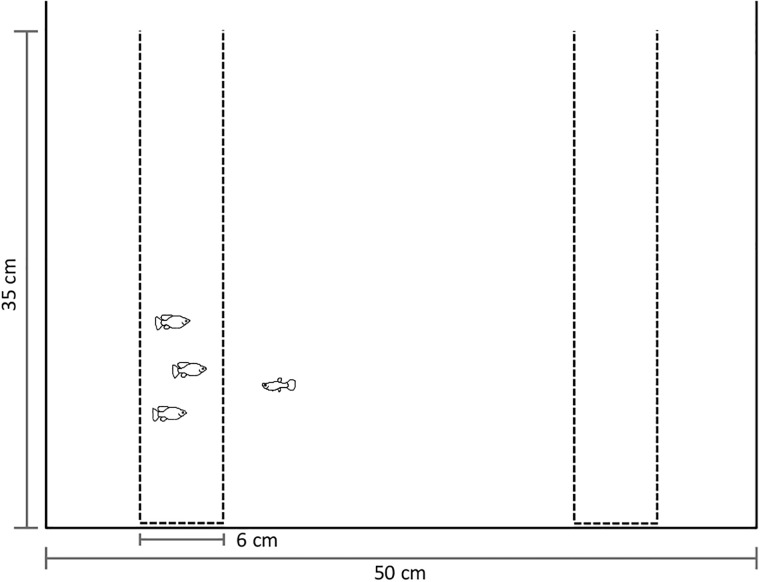
Diagram of the tank set up, one of the bottles contained the given shoal and the other remained empty as a control. Time spent shoaling was recorded whenever the fish was within one body length of the bottle containing a shoal.

Observations were made between 1000 and 1700 h following the methodology used to test tendency by Camacho-Cervantes *et al*. 2014b using a glass tank (50x35x35 cm) and two perforated plastic bottles (6 cm diameter) to allow chemical cues to travel freely in the tank (Fig 1). One bottle contained the shoal and the other remained empty as a control, so we could ensure fish were associating with the shoal contained in the bottle rather than with the bottle itself. Fifteen focal fish of each species were tested, using a repeated measures approach; each fish was presented in a random order with three shoals, one of each species (*P*. *reticulata*, *N*. *bilineata* and *P*. *infans*). Focal fish species and shoals presented to each focal were tested interspaced in time and space to avoid pseudo replication following Hurlbert’s (1984) systematic approach. In between trials, the focal fish was kept in a stand-by tank (20 x 20 x 20 cm) for a period of 60 to 70 min, during which they showed no signs of stressed behaviour. Shoals were formed of three fish taken from stock pools containing between 20 and 28 fish. This was to minimize the probability of pseudoreplication [27]. Observations lasted 10 minutes and were all performed between 10:00 and 13:00 h; association was recorded as occurring whenever the focal fish was within one body length of the bottle containing the shoal. The focal fish was introduced to a third bottle and allowed to settle down for a period of 10 to 15 min, in most cases after the first couple of minutes fish started swimming along de water column and showed no signs of stress. Fish were released by gently lifting and removing this bottle from the tank at this time is when the observation period started. All fish were photographed and standard body length was measured using the image analysis software ImageJ [28]. Each focal fish was used only once. Only females were used to exclude sexually motivated behaviour.

### Ethics statement

Fish were collected under a SEMARNAT-08-049-B permit to collect flora and fauna for research or teaching purposes granted by the Mexican Ministry of Environmental and Natural Resources (SEMARNAT). Fish were transported to the laboratory following the Official Mexican Norm NOM-051-ZOO-1995 for humanitarian treatment in the mobilization of animals. Field and laboratory protocols followed all guidelines provided by the Mexican Official Norm NOM-062-ZOO-1999 for the use and maintenance of vertebrates for research purposes. The Comité de Ética de Investigación Científica (Ethics Committee of the UMSNH) supervised that we followed the applicable Official Mexican Norms. Our experiment included only observational collection of data in the laboratory, thus fish were not sacrificed to preform this study and no fish showed signs of stress during the observational trials.

### Data analysis

In order to control for fish size effects, we used an ANOVA to test if the size of the focal individual relative to that of their test shoal predicted the tendency of fish to associate with guppies, twoline skiffias or Lerma livebearers.

Using one-way t-tests with Holm’s sequential correction (to reduce the chances of incorrectly rejecting a null hypothesis) [29], we tested tendency of fish to join a shoal by comparing the observed duration of the focals in the association zone (area around the bottle where fish were considered to be associated with the given shoal) against the time they would be expected to be in this zone (23s) if they were swimming randomly in the tank. The expected time was calculated using the proportion of the tank volume (61,250 cm^3^) represented by the association zone (2,334.5 cm^3^), which is 3.9%, and calculating this same proportion for the total trial duration (600s). We performed a linear mixed effects model (lme) to evaluate shoaling tendency differences and interactions between focal and shoal species, given that our study uses a repeated measures approach our random factor for the model was the identification number of focals [30, 31]. *A posteriori* Tukey HSD tests (glht) were carried out to identify differences between individual species [32, 33]. All analysis were carried out with the statistical software R [34].

## Results

Size of the focal individual relative to that of their shoal mates was not different between the species of the focal or the shoal (ANOVA, *F*_2,126_< 1.63, *p* > 0.2), nor did it explain any significant proportion of the tendency of fish to associate with guppies (*r*^2^ = 0.004, *p* = 0.28), twoline skiffias (*r*^2^ = 0.001, *p* = 0.34) or Lerma livebearers (*r*^2^ = 0.01, *p* = 0.54). Thus, size was removed from the analysis for the benefit of clarity.

**Fig 2:**
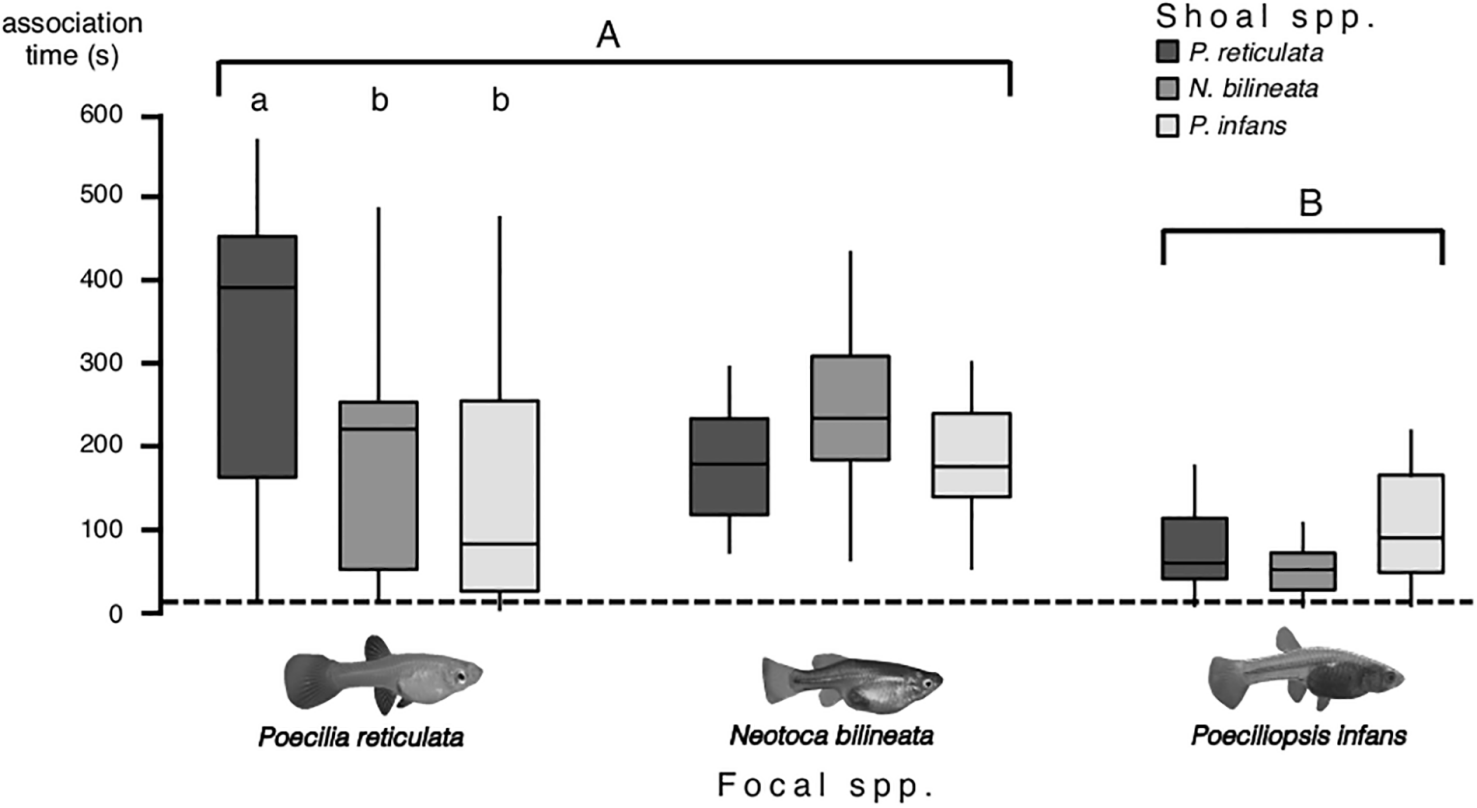
Time (max = 600 s) the focal fish was associated with the given shoal. Horizontal lines in the bars represent the median, boxes indicate interquartile ranges and vertical lines show the range excluding outliers (circles). *P*. *reticulata* and *N*. *bilineata* showed higher sociability than P. infans (uppercase letters). Only *P*. *reticulata* showed differences in its tendency to associate with the species of shoals presented, their tendency to associate with conspecific was higher (lowercase letters).

Fish of all three species spent more time in the proximity of the shoal than would be expected if they were swimming randomly, regardless of the species of fish inside the bottle (one-way *t*-test, t_14_ > 3.26, p < 0.005; after the Holm’s correction p < 0.008). Tendency to associate with other species was different between guppies, twoline skiffias and Lerma livebearer (lme, *F*_2,82_ = 22.25, *p* < 0.001; Fig 2). Post-hoc Tukey HSD test showed that Lerma livebearers are less social than guppies and twoline skiffias (Fig 2). Difference between shoal species was not significant (lme, *F*_2,82_ = 2.06, *p* = 0.132; Fig 2); but there was an interaction between focal species and shoal species (lme, *F*_4,82_ = 4.22, *p* = 0.003; Fig 2). Guppies had a higher tendency to associate with other guppies than with twoline skiffias or Lerma livebearers (lme, *F*_2,11_ = 4.12, *p* = 0.026; Fig 2). Twoline skiffias and Lerma livebearers showed no significant difference in their tendency to associate with the three shoal species (lme, *F*_2,11_ < 3.37, *p* > 0.04; Fig 2), however statistical power for these two test were 47.6% and 48.4% which indicates further test should be carried out to rule out differences due to sample size.

## Discussion

Behavioural adjustments allow invaders to survive in changing environments that present them challenges they do not face in their native habitat [35]. Associations with morphologically similar heterospecifics that share the same ecological requirements allow fish of low density populations to be part of larger groups and potentially avoid Allee effects [23]. Guppies in Trinidad readily shoaled with native poeciliids [24] which indicates that this high sociability can be seen as a pre-existing trait that can facilitate invasion [36]. The present study shows for the first time that not only invasive guppies are willing to shoal with native fish, but that these also prefer to associate with guppies than remaining alone.

Previous research indicates that empty niches and underutilized resources enhance invasion rates [37, 38]. However, our results suggest that ecosystems inhabited by species occupying similar niches to the invader, in this case guppies, can also be at risk of invasion. In addition our data indicate that highly sociable species could overcome the disadvantages of low numbers during early stages of invasion by associating with groups of individuals regardless of the species. Moreover, some native species might even facilitate invasion by not discriminating between other native and invasive shoal partners. This finding follows Simbeloff and Von Holle’s [16] research on interspecific facilitation between invaders leading to an accelerating increase in the number of introduced species and their impact. The difference, and most interesting part, is that our results point to a potential facilitation from a native instead of another invasive species.

Native species might be better at acquiring useful information about surroundings due to their location, use of habitat or specific sensory abilities [39]. The strongest attraction to heterospecifics is expected when the benefits of aggregating with residents exceeds the effects of competition [40]. In the case of goodeids and guppies, it might be that both species benefit during an initial period, but that competition between them could subsequently disadvantage natives [21, 22].

Some mixed species associations occur only for a limited period of time or during certain stages of development. For example, French grunts (*Haemulon flavolineatum*) form mixed schools with at least two species of mysids (genus *Mysidium*). This formation is possible because post larval grunts are morphologically and behaviourally similar to mysids. When postlarval grunts grow, these associations break and grunts benefits trophically from these associations as they prey on the mysids [41]. When benefits depend on traits like similar appearance or similar size, individuals might abandon mixed species shoals when the number of conspecifics decrease [42]. Guppies in our study were the only species showing a significant higher tendency to associate with conspecifics, which lead us to hypothesise that after reaching a certain population size, they might prefer to remain in conspecific shoals. Statistical power of the analysis that compares tendency to shoal with conspecifics and heterospecifics for native species suggest natives might have shown a significant higher tendency to associate with conspecifics had the sample size been larger. However the fact that the sample size was enough to show a significant result for guppies, suggests that guppies do show a higher tendency to associate with conspecifics than native species.

Interactions between native and exotic species have been described in many taxa [43–45]. For example, a study carried out in Florida showed that the resistance to be invaded by native species is reducing the success of introduced fishes; the eastern mosquito fish (*Gambusia holbrooki*) attacked and killed non-native poeciliids (*Xiphophorus variatus* and *Xiphophorus hellerii)* [46]. In the latter study, the negative effects on the non-native poeciliids were higher when gambusias were more abundant. Contrary to these results, we found guppies could be joining heterospecific shoals through mutual shoaling choices and benefit from native’s familiarity with the environment. However, we acknowledge that our experimental design does not allow us to test for true agonistic or cooperative behaviours and further research should be done using an experimental design that allows fish to interact freely.

Invasion success depends on finding a time or place where invaders are able to establish and in some cases outweigh resident species [47]. Native Mexican topminnows might be providing this time and place to invasive guppies by being willing to associate with them during the critical initial stages of invasion. Environments where native species could facilitate invaders to reach a viable population and establish are thus under a higher risk of invasion.

## Supporting information

S1 Table Mutual shoaling choices. Data set including the records of association tendency for each focal of the three tested species (*P*. *reticulata*, *P*. *infans* and *N*. *bilineata*) towards the species of shoals presented (*P*. *reticulata*, *P*. *infans* and *N*. *bilineata*).
(TXT)
